# Characteristics and Genomic Diversity of Measles Virus From Measles Cases With Known Vaccination Status in Shanghai, China

**DOI:** 10.3389/fmed.2022.841650

**Published:** 2022-06-30

**Authors:** Xiaoxian Cui, Yunyi Li, Yuying Yang, Wei Tang, Zhi Li, Hongyou Chen, Yang Li, Xinyi Cui, Zhuoying Huang, Xiaodong Sun, Songtao Xu, Yan Zhang, Chongshan Li, Xi Zhang

**Affiliations:** ^1^Division of Microbiology, Shanghai Municipal Center for Disease Control and Prevention, Shanghai, China; ^2^Department of Immunization Program, Shanghai Municipal Center for Disease Control and Prevention, Shanghai, China; ^3^Chongqing School, University of Chinese Academy of Sciences, Chongqing, China; ^4^National Health Commission (NHC) Key Laboratory of Medical Virology and Viral Diseases, Chinese Center for Disease Control and Prevention, Beijing, China

**Keywords:** measles virus, complete genome sequences, Shanghai isolates, genetic diversity, vaccine failure, comparative genomics

## Abstract

Although the highly effective measles vaccine has dramatically reduced the incidence of measles, measles, and outbreaks continue to occur in individuals who received the measles vaccine because of immunization failure. In this study, patients who have definite records of immunization were enrolled based on measles surveillance in Shanghai, China, from 2009 to 2017, and genomic characteristics regarding viruses retrieved from these cases provided insights into immunization failure. A total of 147 complete genomes of measles virus (MV) were obtained from the laboratory-confirmed cases through Illumina MiSeq. Epidemiological, and genetic characteristics of the MV were focused on information about age, gender, immunization record, variation, and evolution of the whole genome. Furthermore, systematic genomics using phylogeny and selection pressure approaches were analyzed. Our analysis based on the whole genome of 147 isolates revealed 4 clusters: 2 for the genotype H1 (clusters named H1-A, including 73 isolates; H1-B, including 72 isolates) and the other 2 for D8 and B3, respectively. Estimated nucleotide substitution rates of genotype H1 MV derived using genome and individual genes are lower than other genotypes. Our study contributes to global measles epidemiology and proves that whole-genome sequencing was a useful tool for more refined genomic characterization. The conclusion indicates that vaccination may have an effect on virus evolution. However, no major impact was found on the antigenicity in Shanghai isolates.

## Introduction

Measles is caused by the measles virus (MV), which is one of the most contagious diseases of humans before introducing the measles vaccine. MV is a member of the genus Morbillivirus of the family Paramyxoviridae (1). The virus contains a negative-sense RNA genome of 15,894 nucleotides that consists of 6 structural proteins: nucleoprotein (N), phosphoprotein (P), matrix (M), fusion (F), hemagglutinin (H), and large protein (L), and 2 additional non-structural proteins, C and V. Although MV is monotypic, it is divided into 8 clades and 24 genotypes based on the nucleotide sequences of the N terminal 450 (N-450) that encode the C terminal region of the protein as the main target for genotyping (2, 3) and the complete coding sequence of H genes of about 1845 nucleotides as a complementary target (4), according to the WHO Global Measles and Rubella Laboratory Network’s (GMRLN) guidelines.

Since 1978, the live attenuated measles vaccine has been included in the National Expanded Program on Immunization (EPI) plan. In Shanghai, the vaccination procedure for the 2 dose was changed from 8 months and 7 years between 1986 and 2004 (5) to 8 and 24 months in 2005. The widespread use of vaccines belonged to genotype A. However, given that the measles cases were imported from varying regions with different measles vaccine strategies, those viruses currently circulating in Shanghai, China were classified as genotype H1 (sometimes B3 and D8) (6). The re-emergence is a major concern, especially considering the vaccinated individuals.

In view of the WHO being set up as a target for endorsing the Western Pacific Regional Plan of Action for Measles Elimination (7), a high level of routine immunization coverage and a well-performing surveillance system, including highly sensitive laboratory and high-quality epidemiological assessments, need to be strengthened. In addition, molecular characterization and phylogenetic analyses of MV were essential to monitor the progress of measles immunization and elimination efforts. Studies on MV were mostly based on either an analysis of genomes pertaining to individual groups such as wild-type and vaccine or on an analysis based on single genes such as H, F, and N (8–11) in the past. Besides, with the development of Next Generation Sequencing (NGS), studies about variation and molecular evolution based on complete genomes were performed in a few recent reports (12–16). Furthermore, a number of reports evaluated and discussed the characterization of vaccinated cases (5, 17). Unfortunately, those studies did not include the complete genomes of MV from immune failure cases. In this study, our aim was to establish an MV-sequencing platform using the NGS Illumina MiSeq. After obtaining the whole genome MV sequences from measles cases with a clear history of vaccination (*n* = 147), we next performed an in-depth analysis to understand the genetic characteristics of MV.

## Materials and Methods

### Ethics Statement

The experiments and protocols in this study were screened and approved by the Ethical Review Committee of the Shanghai Municipal Centre for Disease Control and Prevention (Shanghai CDC). Clinical specimens, including throat swabs and sera, were obtained from the routine MV surveillance program of the Shanghai CDC. Sample collection was approved by either the patients or their parents with prior informed consent.

### Measles Surveillance System and Case Definition

The demographic data of the patients were obtained from the China Information System for Disease Control and Prevention (CISDCP). The definition of suspected measles cases was based on the guidelines of the Shanghai Municipal Measles and Rubella Surveillance Program, confirmed by the WHO case definition for measles (fever, generalized maculopapular rash, and cough, coryza, or conjunctivitis). A laboratory-confirmed case was defined by serological (positive serologic test for measles IgM antibody or fourfold rise in measles IgG by standard serologic assay) or virological (identification of the virus RNA by RT-PCR or isolation of the virus from a clinical specimen) evidence of acute measles infection according to the MMS. Measles immunization has been documented since 2009 when the measles surveillance system (MSS) was established. The vaccination statuses of all cases in our study were determined by their vaccination card or memories of patients.

### The Sample Collection and Isolation of the Measles Virus Strains

The throat swab samples in this study were collected according to the MSS in Shanghai. This study strictly followed the relevant ethical requirements and strictly protected the data security and privacy of the participants. Clinical samples that were positive by PCR were inoculated on cell culture to isolate the virus. The Vero/hSLAM cell line was used for MV isolation from clinical specimens. Vero/hSLAM cells were kept in our laboratory and kindly shared by Dr. Yanagi at Kyushu University, Japan, through the WHO GMRLN. Procedures including the maintenance of cells, sample handling, and virus inoculation were performed as described in the Manual for the Laboratory-Based Surveillance of Measles, Rubella, and Congenital Rubella Syndrome (WHO).

### Whole-Genome Sequencing

The virus genomes of the isolated MV strains were extracted and purified using the QIAamp Viral RNA Mini Kit (Qiagen, Valencia, CA, United States). The whole genome was amplified with four overlapping long PCR fragments (3.9–5.4 kb) using the high-fidelity enzyme mixture of the SuperScript III One-Step RT-PCR Kit (Invitrogen, Carlsbad, CA, United States) as described in a previous study (18, 19) and sequenced using the Illumina MiSeq platform (9885 Towne Center Drive, San Diego, CA, United States) and the MiSeq Reagent Kit version 3 (600-cycles), according to the manufacturer’s protocols. Paired-end libraries for the MiSeq platform were prepared based on Nextera XT DNA Library Preparation Kit (24 samples). Low-quality reads were removed, and reads were mapped on representative MV genomes using CLC Genomics Workbench software (Hilden, Germany). Consensuses were extracted after the read-mapping process by removing the regions not covered and joined. In addition, we applied vote resolution to handle conflicts, considering both average coverage and consensus sequence length.

### Genetic Variability Assay of Full-Length Sequences

The nucleotide variability of the whole genome was calculated *via* pairwise distance mapping using SSE, version 1.2 England, University of Oxford (20). The scan used the group and fragment lengths of 250 base pairs (bp) at 50-bp increments across the whole genome. The genetic distances (substitution/site) were calculated using *p*-distance in the MEGA program. This distance indicates the proportion (p) of nucleotide/amino acid sites at which being compared are different. It is obtained by dividing the number of nucleotide/amino acid differences by the total number of nucleotides compared.

### Genotyping Analysis

For the genotyping step of MV isolates, the nucleotides and amino acid sequences were aligned using the MEGA program (version 6 and X) (Sudhir Kumar, Arizona State University, Tempe, AZ, United States). Phylogenetic trees were constructed by the maximum likelihood method with the best model implemented in the MEGA program. The reliability of phylogenetic inference at each branch node was estimated by the bootstrap method with 1000 replications. Bootstrap values greater than 70% were considered statistically significant for grouping. The nucleotide substitution rates were estimated by a coalescent-based Bayesian method in BEAST (version 2.6.0)^1^. The Simplot program (version 3.5.1, Stuart Ray, John Hopkins University, Baltimore, MD, United States) was used for the recombination analysis. The included strains represent the Shanghai MV strains isolated in comparison with reference strains defined by the WHO. Phylogenetic reconstruction was conducted on the nucleoprotein peptide sequences of these strains, as recommended by the WHO for genotype. The same phylogeny was established for the individual genes N, M, F, and H.

### Selection Pressure Studies

Gene sequences of 147 isolates were used for the selection pressure analysis by the Datamonkey server. Codon-based multiple sequence alignments of an individual gene were carried out using the MUSCLE program available in the MEGA X. The ratio of non-synonymous to synonymous substitutions per site (dN/dS) at every codon was calculated using Single-Likelihood Ancestor Counting (SLAC) and Fixed Effects Likelihood (FEL) methods with *p*-value ≤ 0.05.

## Results

### Demographic and Vaccination Parameters From 2009 to 2017

Since 2001, high-quality pathogenic measles surveillance has been maintained in Shanghai. From 2009 to 2017, the measles incidence in Shanghai was between 0.2/100,000 and 3.82/100,000 annually. According to the requirements of the national measles surveillance program, throat swabs are required to be collected for MV separation for every suspected measles case since 2011. A total of 3465 measles cases were confirmed in Shanghai by the laboratory using MV IgM testing or MV RNA testing by real-time RT-PCR assay. Furthermore, 1204 strains were isolated from these cases. According to the MSS, 1288 were unvaccinated cases and 296 cases were vaccinated (shown in Supplementary Figure 1A). In our study, 147 isolates with a definite vaccination history were included, among which 98 (98/147, 66.7%) were unvaccinated patients whereas 39 (39/147, 26.5%) and 10 (10/147, 6.8%) had received 1 and more than 1 dose of vaccine, respectively (shown in Supplementary Figure 1B). The sexual and age group distribution and vaccine status data of these cases were shown in Table 1, which were observed in the ages from 4 months to 57 years old. Furthermore, a total of 39 cases were infants under the age of 8 months (before the recommended age of the first vaccine dose) between unvaccinated cases.

**TABLE 1:** The demographic characteristics of measles isolate included in the study (*n* = 147), Shanghai, 2009–2017.

Characteristics	Measles vaccination status	
	MCV ≥ 1 (%)	MCV = 0 (%)
**Sex**		
Male	32 (65.3)	63 (64.3)
Female	17 (34.7)	35 (35.7)
**Age group**		
0–7 months	1 (2.0)	39 (39.9)
8–11 months	17 (34.7)	25 (25.5)
1–9 years	14 (28.6)	16 (16.3)
10–19 years	7 (14.3)	2 (2.0)
20–29 years	8 (16.3)	5 (5.1)
30-years	2 (4.1)	11 (11.2)
Total	49	98

### The Routine Genotype of the Measles Virus Isolates

**FIGURE 1:**
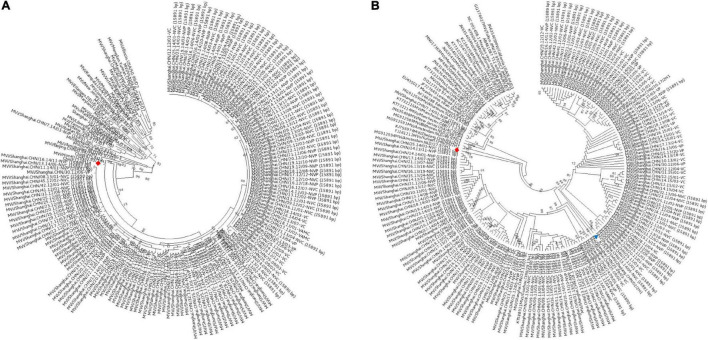
Genotyping of MV strains circulating in Shanghai, China, between 2009 and 2017. **(A)** Based on the full-length 150 amino acid sequence of the N terminal 450 bp in the nucleoprotein region; **(B)** H gene. A maximum-likelihood phylogenetic reconstructed by MEGA 6 was performed with 147 Shanghai-MV strains and 24 genotype-references from the WHO’s list for genotyping. ◆: reference strains from genotype H1; ▲: reference strains from clusters H1-A.

In this study, 147 complete MV genome sequences were successfully generated from the MV isolates and planned to submit to GenBank. Routine phylogenetic analyses of the carboxyl-terminal 150 amino acids encoded by the N-450 hypervariable region of the nucleoprotein gene, which was shown in Figure 1A, which indicated that these isolates mostly belong to the genotypes H1a. The genotype H1 was predominant with 145 isolates cluster with Chin9322/H1a, whereas only 2 isolates, Mvi/Shanghai.CHN/40.12/01-VC and Mvi/Shanghai.CHN/7.14/01-NVP belonged to the genotype D8 and B3, respectively, the phylogenetically closest reference sequences being Mvi/Manchester.GBR/30.94/D8 and Mvi/Ibadan.NGA/0.97/1/B3, with a bootstrap value of 99. The other H1a isolates (sequenced in this study) were hard to determine the lineages because of the low bootstrap values. The genotyping result of the H gene (Figure 1B) was consistent with the N-450.

### Genomics Variability and Comparative Analysis of the Measles Virus Isolates by Patients’ Vaccine Status

**FIGURE 2:**
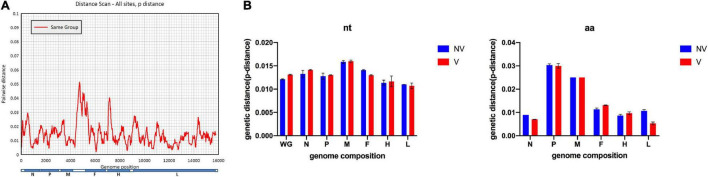
Comparison of the vaccine group and non-vaccine group for characterizing the measles virus genome. **(A)** Pairwise distance based on 147 full-length measles virus sequences. **(B)** The estimated average divergence in individual genes is based on nucleotide (nt) sequences or predicted amino acid (aa) sequences.

A scan of sequence variability across the full MV genome is shown in Figure 2A, which identified an MF-HVR region between the M and F genes as the most variable gene region, which was consistent with previous reports (21), with a mean pairwise distance of 0.035 (range, 0.002–0.16). Nucleotide divergence observed among Shanghai MV isolates was 0.001–0.074. The overall mean pairwise distance is 0.014. The mean divergence values of the H-gene and N-450 region of the isolates are 0.008 and 0.01 in B3 isolates and 0.013 and 0.025 in D8 isolates, respectively. Complete genome, as well as individual gene and protein sequence data of all the isolates, were compared with sequences of vaccine strains. The extent of sequence similarity for complete genomes was observed to be 0.94–0.96. Sequence similarity values (%) for both gene and protein sequences are N: (∼92.1–99.7; ∼94.7–96.3); F: (∼94.1–96.6; ∼95–97); M: (∼97.2–99.3; ∼98.7–99.5); H: (∼93.6–96.8; ∼98.4–99.3); P: (∼94.3–96.5; ∼93.3–96.1); L: (∼94.1–96.2; ∼97.4–98.9), respectively.

Furtherly, sequences divided into two groups by vaccine status were analyzed. Sequence similarity including nucleotide and amino acid of the whole genome and individual genes were calculated and analyzed, as shown in Figure 2B. In general, group V (vaccine) is more diverse than group NV (non-vaccine). The genetic diversity within group V is 0.014; while it is 0.013 within the group NV based on the whole genome sequences.

### Phylogenic Analysis of Measles Virus Genomes Using Complete Genomic Sequences and Different Genes

**FIGURE 3:**
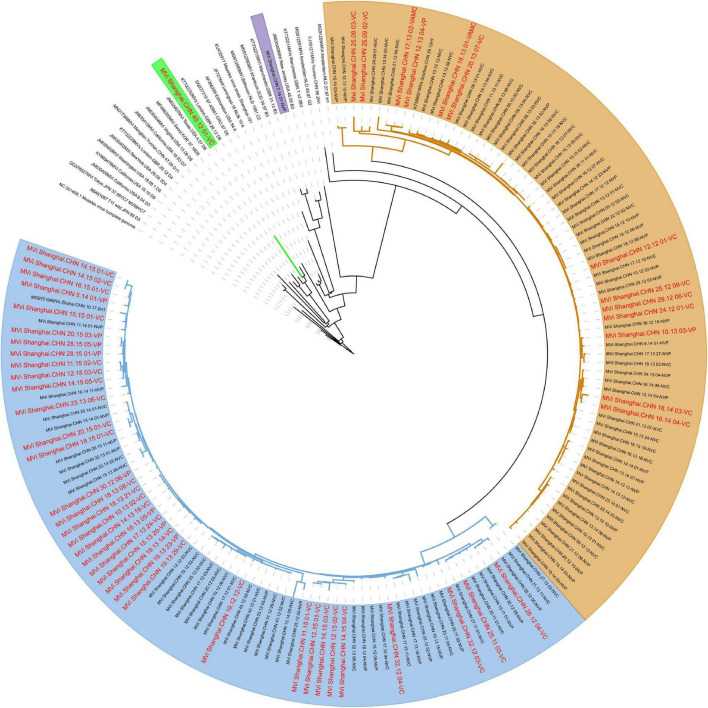
Maximum clade credibility tree of 147 Shanghai and global MV isolates derived using the whole genome. The D8, B3, and H1-A, H1-B are highlighted using green, purples, yellows, and blues, respectively.

The evolutionary relaSAtionships based on the whole genome sequences between all the isolates revealed 4 clusters: 2 for the genotype H1 (clusters named H1-A, including 73 isolates; H1-B, including 72 isolates) and the other 2 for D8 and B3 (Figure 3). Although some small lineages formed, no more clusters formed because of low bootstrap values. Further analysis found that the phylogenetically closest reference sequence was Mvi/Zhengjiang.CHN/02/2H1 (accession number: KJ755974). Interestingly, the virus isolated from the vaccinated cases accounted for 50% in cluster H1-A and 20% in cluster H1-B. This clustering pattern was also observed when independent trees were generated using single genes H, as well as MF-HVR (Supplementary Figure 2), yet not genotype of N-450. Furthermore, the evolutionary relationships based on genes F and M are shown in Supplementary Figures 3, 4.

### The Evolutionary Rate of Measles Virus and Recombination

Measles isolates were found to be devoid of recombination in the current study (16). The general time-reversible (GTR) model + proportion of invariable sites (I) + gamma distribution shape parameter (G) substitution-strict clock model with a constant growth rate was chosen as the acceptable model based on AICM values. As can be seen from Table 2, the NSRs obtained using complete genomes were higher than that reported about the global isolates for other genotypes. NSR of individual genes *viz*., N, H, and F were also calculated. Varying NSRs observed for N-, H-, and F-genes are 7.92 × 10^–4^ (95% HPD: 5.74 × 10^–4^, 1.01 × 10^–3^), 8.43 × 10^–4^ (95% HPD: 6.47 × 10^–4^, 1.05 × 10^–3^), and 8.83 × 10^–4^ (95% HPD: 6.94 × 10^–4^, 1.08 × 10^–3^) substitutions/site/year, respectively (Table 2). The dN/dS ratio across individual genes of strains in our study calculated using SLAC when arranged in their decreasing order is H (0.37), P (0.35), F (0.33), N (0.27), M (0.22), and L (0.21).

**TABLE 2:** Nucleotide substitution rates of MV isolates and individual genes in Shanghai.

Group	Nucleotide substitution rate (substitutions/site/year)	95% HPD	ESS
Shanghai isolates whole genome	5.31 × 10^–4^	4.53 × 10^–4^, 6.03 × 10^–4^	197
Group V	5.81 × 10^–4^	4.76 × 10^–4^, 6.80 × 10^–4^	585
Group NV	3.47 × 10^–4^	2.76 × 10^–4^, 4.27 × 10^–4^	221
Gene N	7.92 × 10^–4^	5.74 × 10^–4^, 1.01 × 10^–3^	385
Gene H	8.93 × 10^–4^	6.47 × 10^–4^, 1.05 × 10^–3^	212
Gene F	8.82 × 10^–4^	6.94 × 10^–4^, 1.08 × 10^–3^	296

### Selection Pressure Analysis of Shanghai Isolates and Structure-Function Correlation of Mutations Observed in H-Protein

Selection pressure analysis was carried out for individual genes, and in genes P, M, F, H, and L, a few codons were observed to be under selection, and their functional implications which were reported are listed in Table 3.

**TABLE 3:** Gene-wide distribution of codons under pervasive selection.

Gene	Codons	Functional role
P	30, 61	Amino acid Glu-30 is a part of cytotoxic T-cell epitope (37)
N	136, 422, 497	No related functional study was reported
H	493, 592, 603	Amino acid 493 and 603 were hypervariable sites reported by unpublished data from the Chinese Center for Disease Control and Prevention

*No codons have been found under selection in F, L, and M genes.*

The H glycoprotein is the major target for neutralizing antibodies directed against the virus and binding to host receptors like signaling lymphocyte-activation molecule (SLAM) and Nectin-4. Furthermore, H-protein is known to be glycosylated at amino-acid positions 168, 187, 200, 215, and 238, whereas position 416 is an additional potential N-linked glycosylated site. It is found to be completely conserved for these function sites in Shanghai isolates, and none of the amino acids that are encoded by the codons under selection in H-gene are part of the known function sites.

## Discussion

China established a National EPI in 1978. Until 2010, after more than 30 years of routine and synchronized nationwide supplementary immunization activities (SIA) (22), the incidence of measles in China has decreased significantly since 1986 and had been maintained at a very low level (23–25). Although a measles epidemic caused a resurgence from 2014 to 2016 (26), in Shanghai, the incidence and the scale of the outbreak continued to rise from 2012 to 2015 (27, 28). According to our investigation, most measles infections were not vaccinated, and analyses of the demographics of the measles revealed immunity gaps in children under the age of 9 and adults over the age of 30.

To evaluate the utility and scope of the whole genome sequencing, the WHO GMRLM have formed the “Next-generation. Extended window and Whole genome sequencing working group” (N.E.W.) (29), which described the current needs and applications of whole-genome sequencing for measles and rubella surveillance. However, a limited number of MV complete genome sequences are available in GenBank, especially for genotype H1.

For the first time, our study evaluated the genetic diversity and evolutionary dynamics of 147 MV isolates circulating in Shanghai from 2009 to 2015 based on whole-genome sequence analyses, which is a significant addition to MV global data. Thus, this study was conducted to establish the genetic and epidemiological characteristics of the measles cases with immunization records in Shanghai between 2009 and 2017. The virological characteristics of these isolates from these infections are reported for the first time.

According to the routine genotyping in our report, only three genotypes have been identified in these cases since 2009, and the most prevalent were H1a and D8 in 2008 and B3 in 2009, which have also been in circulation since the molecular measles surveillance was established in 2001 (27). However, it has become increasingly difficult to determine the origin and evolutionary relationship among these strains from the same genotype, as the genetic variability of circulating virus strains decreased (22). Therefore, the genotype-based clustering observed using the whole genome phylogeny of 147 Shanghai and 38 global isolates suggests the existence of at least 2 clusters of H1 and other lineages in Shanghai. The lack of monophyletic lineages based on geographical regions in our exploration suggests the spread of measles cases that can be associated with travel. Our study proves that genotype is not an influencing factor for breaking the vaccine barrier, and more sensitive monitoring systems and vaccination rates are needed. Recombination in MV based on the analysis of the complete genome was lack of evidence. Furthermore, no insertions and deletions (indels) were found for the H1 isolates in our study. D4 isolates from India (16) and the United States (30) were observed with non-standard lengths.

The evolutionary rates of MV in different genotypes based on single genes like H and N are variable, ranging from 7.28 × 10^–6^ substitutions/site/year to 6 × 10^–3^ substitutions/site/year (31, 32). We estimated the nucleotide substitution rate of genotype H1 based on the different genes. The estimate of the evolutionary rate of each sub-genotype also found that sub-genotypes H1-A and H1-B exhibited a similar rate, while group nv had the slowest evolutionary rate. Additionally, compared with the evolutionary rate of the H and F genes, the N gene evolved most slowly but faster than the whole genome. Thus, the rate of genome-wide NSRs compared to other genotypes hints at different evolution of MV in China. However, it must be mentioned that variations in sample size, time, and place of isolation are known to impact the estimation of substitution rates. The overall comparison of genome-wide NSR is indicative of constraints on the evolution of MV as compared to other RNA viruses in general, such as enterovirus 71 (3.18 × 10^–3^), human influenza A (1.8 × 10^–3^), and slightly higher than the mutation rates reported for dengue virus and human rotavirus (33, 34).

We tried to find whether there was a difference between group v and group nv at the genome level through genome-wide association study (GWAS). However, there was no significant result. In the selection pressure analysis, no differences were found. The values of NSRs and dN/dS ratios correlate with the structural, functional, and evolutionary constraints operational on these genes. The observed dN/dS ratios also hint at the modest role of immune selection in MV evolution, wherein the highest variability was observed in H-gene, the major target for neutralizing antibodies directed against the virus and binding to host receptors. The lowest ratio was observed in the case of L-gene that encodes RNA-dependent-RNA-polymerase which has been reported to be most refractive to insertional mutagenesis. Thus, complete genome sequencing data of 147 isolates sampled during 2009–2017 substantially increment global genomic data of genotypes H1 isolates. The genetic diversity of China Shanghai isolates was found to be moderate in our report, and our study also underlines constraints on the evolution of MV with no potential impact on antigenicity. This knowledge is valuable for policy decisions regarding MV surveillance and control in the eliminating countries and the areas of measles.

Our study, based on viral isolates, provides for the reintroduction of isolation-on-cell-culture as a useful tool for the characterization and comprehensive study of the molecular epidemiology of circulating strains of MV. However, there were also disadvantages, like single nucleotide polymorphisms may be obtained less than directly from clinical samples. Furthermore, amplification to obtain whole genome in our experiment may cause unnatural mutations in the virus genomes. In fact, the high cost made it not applicable on a routine basis in all laboratories (35). Due to the COVID-19 epidemic and the continuous decline in sequencing costs, the development of MV whole-genome sequencing by NGS appears to have the potential for the future and needs to be implemented more widely in routine diagnostic laboratories.

## Data Availability Statement

The original contributions presented in this study are publicly available. This data can be found here: BankIT, ON035847–ON035993.

## Ethics Statement

The experiments and protocols in this study were screened and approved by the Ethical Review Committee of the Shanghai Municipal Centre for Disease Control and Prevention (Shanghai CDC). Clinical specimens, including throat swabs and sera were obtained from the routine measles virus surveillance programme of the Shanghai CDC. Sample collection was approved by either the patients or their parents with prior informed consent.

## Author Contributions

XZ and CSL designed this study and revised the manuscript. XXC sequenced the samples, analyzed the data, and wrote this manuscript. WT, YYL, YY, and XYC contributed to sample testing. HYC and YL analyzed the data. ZL, YZ, and XDS collected the samples and revised the manuscript. STX and YZ contributed to the design of this study. All authors approved the final manuscript as submitted and agreed to be accountable for all aspects of the work.

## Conflict of Interest

The authors declare that the research was conducted in the absence of any commercial or financial relationships that could be construed as a potential conflict of interest.

## Publisher’s Note

All claims expressed in this article are solely those of the authors and do not necessarily represent those of their affiliated organizations, or those of the publisher, the editors and the reviewers. Any product that may be evaluated in this article, or claim that may be made by its manufacturer, is not guaranteed or endorsed by the publisher.

## Footnotes

1
https://beast.community/index.html
