# Supplementary material for: Characteristics and Genomic Diversity of Measles Virus From Measles Cases With Known Vaccination Status in Shanghai, China
Source: Front Med (Lausanne). 2022 Jun 30;9:841650. doi: 10.3389/fmed.2022.841650 (PMC9281471; doi:10.3389/fmed.2022.841650)
Supplement: Supplementary file 1 [file Data_Sheet_1.pdf]

Supplementary Material

1 Supplementary Figures and Tables

1.1 Supplementary Figures

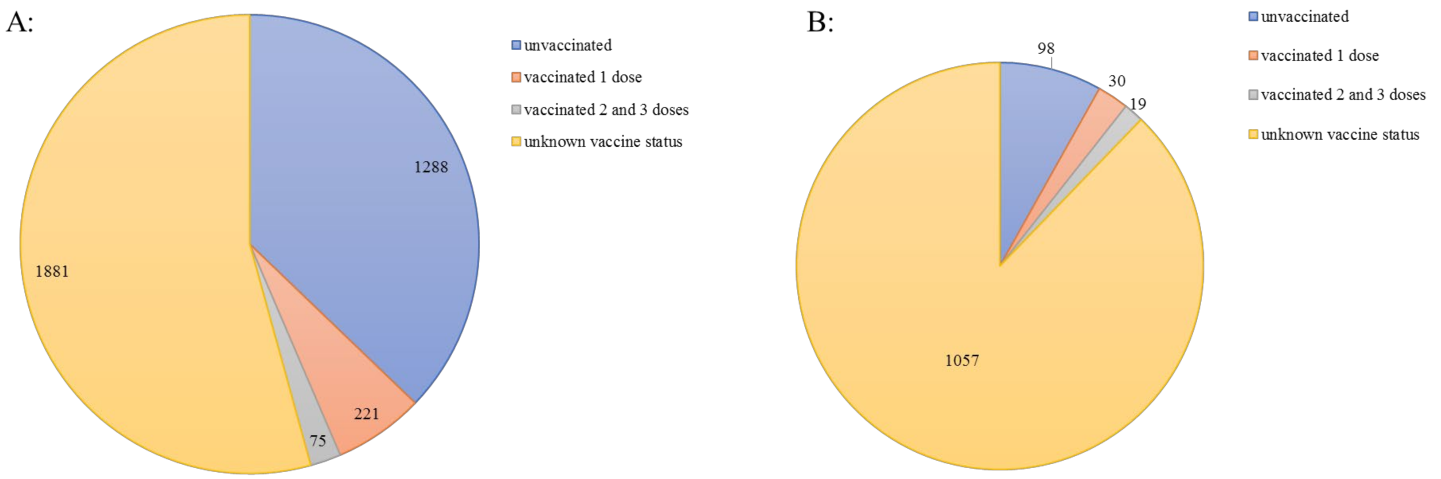

Supplementary Figure 1. Numbers of laboratory-confirmed measles cases (Left A, N=3465) and virus isolates (Right B, N=1204) by vaccination status.

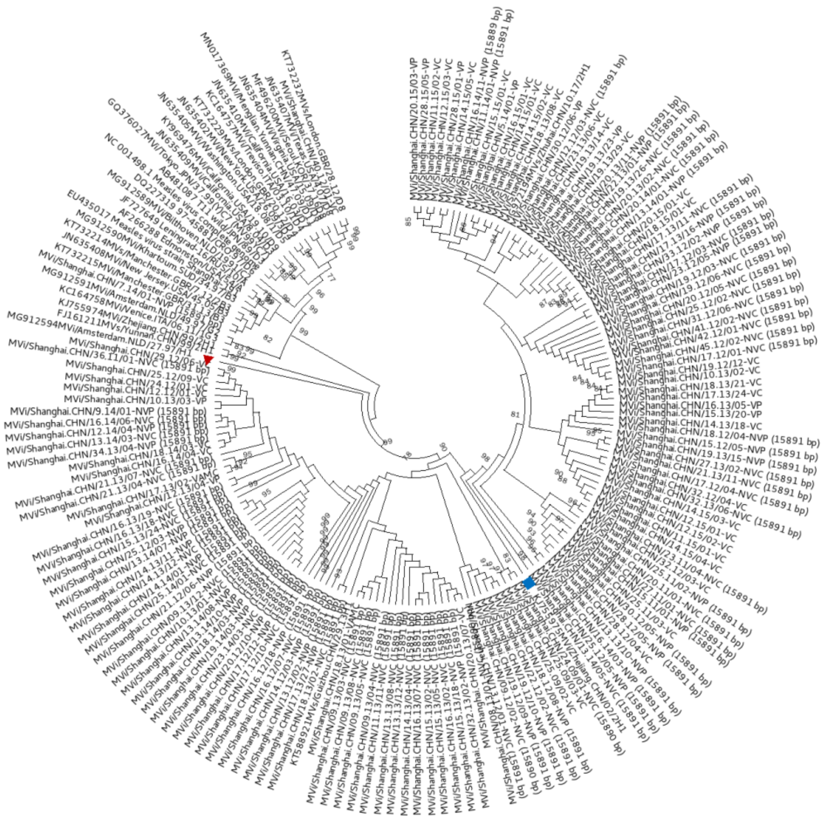

Supplementary Figure 2. Genotyping of MV strains circulating in Shanghai, China, between 2009 and 2017 based on the MF-HVR region between the M and F genes as the most variable gene region. A maximum likelihood phylogenetic reconstructed by MEGA 6, was performed with 147 Shanghai-MeV strains and the 24 genotype-references for genotyping. ▼: reference strains from genotype H1; ▲: reference strains from clusters H1-A.

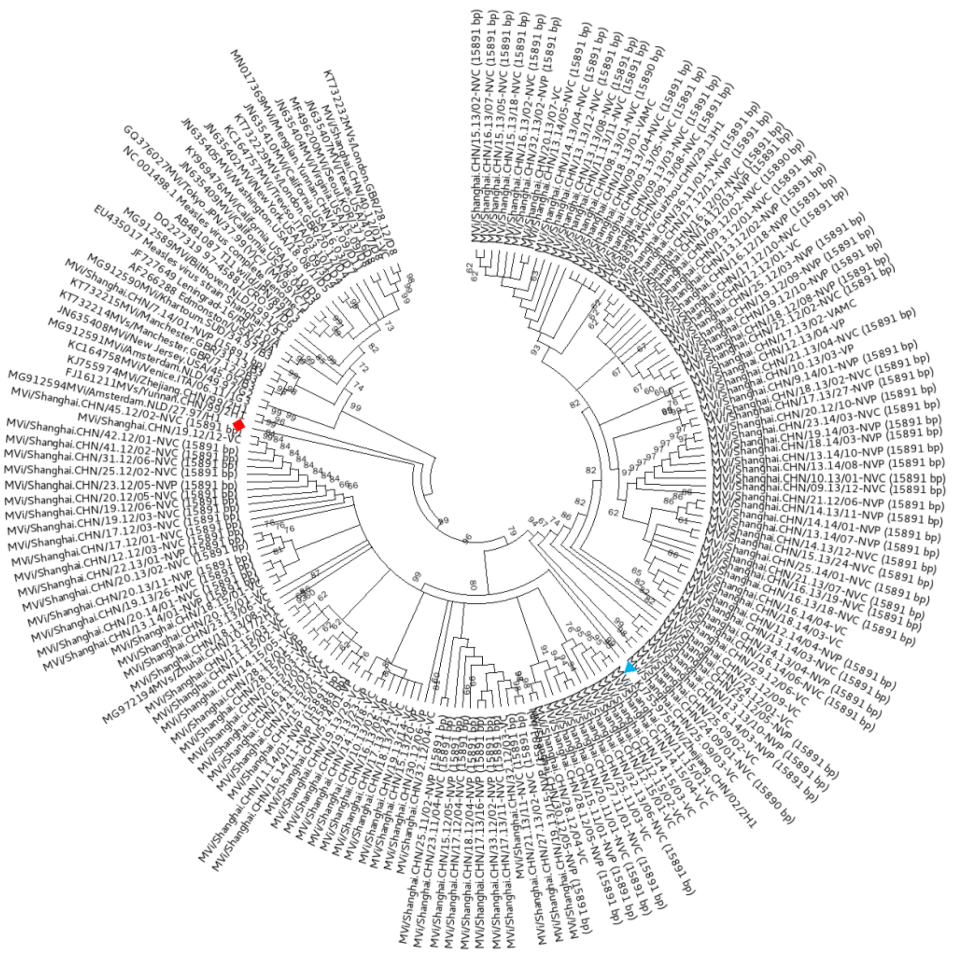

Supplementary Figure 3. Genotyping of MV strains circulating in Shanghai, China, between 2009 and 2017 based on the gene F. A maximum likelihood phylogenetic reconstructed by MEGA 6, was performed with 147 Shanghai-MeV strains and the 24 genotype-references for genotyping. ▼: reference strains from genotype H1; ▲: reference strains from clusters H1-A.

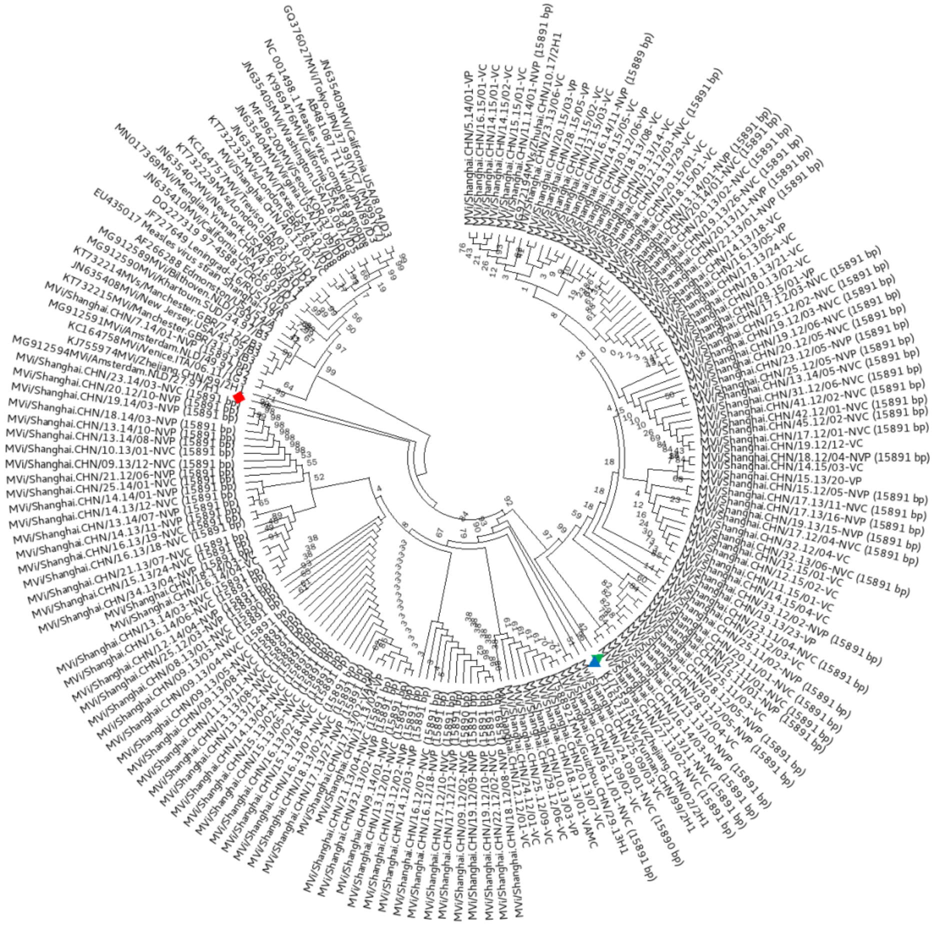

Supplementary Figure 4. Genotyping of MV strains circulating in Shanghai, China, between 2009 and 2017 based on the gene M. A maximum likelihood phylogenetic reconstructed by MEGA 6, was performed with 147 Shanghai-MeV strains and the 24 genotype-references for genotyping. ▼: reference strains from genotype H1; ▲: reference strains from clusters H1-A.
